# Antiparasitic mebendazole (MBZ) effectively overcomes cisplatin resistance in human ovarian cancer cells by inhibiting multiple cancer-associated signaling pathways

**DOI:** 10.18632/aging.203232

**Published:** 2021-07-07

**Authors:** Linjuan Huang, Ling Zhao, Jing Zhang, Fang He, Hao Wang, Qing Liu, Deyao Shi, Na Ni, William Wagstaff, Connie Chen, Russell R. Reid, Rex C. Haydon, Hue H. Luu, Le Shen, Tong-Chuan He, Liangdan Tang

**Affiliations:** 1 Department of Obstetrics and Gynecology, The First Affiliated Hospital of Chongqing Medical University, Chongqing, China; 2 Molecular Oncology Laboratory, Department of Orthopaedic Surgery and Rehabilitation Medicine, The University of Chicago Medical Center, Chicago, IL 60637, USA; 3 Department of Medicine/Gastroenterology, The First Affiliated Hospital of Chongqing Medical University, Chongqing, China; 4 Ministry of Education Key Laboratory of Diagnostic Medicine, and the School of Laboratory Diagnostic Medicine, Chongqing Medical University, Chongqing, China; 5 Department of Spine Surgery, Second Xiangya Hospital, Central South University, Changsha 410011, China; 6 Department of Orthopaedic Surgery, Union Hospital of Tongji Medical College, Huazhong University of Science and Technology, Wuhan 430022, China; 7 Department of Surgery Section of Plastic Surgery, The University of Chicago Medical Center, Chicago, IL 60637, USA; 8 Department of Surgery, The University of Chicago Medical Center, Chicago, IL 60637, USA

**Keywords:** ovarian cancer, cisplatin, chemoresistance, cisplatin resistance, mebendazole (MBZ), drug repurposing

## Abstract

Ovarian cancer is the third most common cancer and the second most common cause of gynecologic cancer death in women. Its routine clinical management includes surgical resection and systemic therapy with chemotherapeutics. While the first-line systemic therapy requires the combined use of platinum-based agents and paclitaxel, many ovarian cancer patients have recurrence and eventually succumb to chemoresistance. Thus, it is imperative to develop new strategies to overcome recurrence and chemoresistance of ovarian cancer. Repurposing previously-approved drugs is a cost-effective strategy for cancer drug discovery. The antiparasitic drug mebendazole (MBZ) is one of the most promising drugs with repurposing potential. Here, we investigate whether MBZ can overcome cisplatin resistance and sensitize chemoresistant ovarian cancer cells to cisplatin. We first established and characterized two stable and robust cisplatin-resistant (CR) human ovarian cancer lines and demonstrated that MBZ markedly inhibited cell proliferation, suppressed cell wounding healing/migration, and induced apoptosis in both parental and CR cells at low micromole range. Mechanistically, MBZ was revealed to inhibit multiple cancer-related signal pathways including ELK/SRF, NFKB, MYC/MAX, and E2F/DP1 in cisplatin-resistant ovarian cancer cells. We further showed that MBZ synergized with cisplatin to suppress cell proliferation, induce cell apoptosis, and blunt tumor growth in xenograft tumor model of human cisplatin-resistant ovarian cancer cells. Collectively, our findings suggest that MBZ may be repurposed as a synergistic sensitizer of cisplatin in treating chemoresistant human ovarian cancer, which warrants further clinical studies.

## INTRODUCTION

Ovarian cancer (OC) is the third most common cancer and the second most common cause of gynecologic cancer death in women globally [1–6]. Due to the lack of effective early detection strategies, over 80% of ovarian cancers are usually diagnosed with metastatic lesions. In fact, ovarian cancer is three times more lethal than breast cancer even though it has a lower prevalence [1, 4–8]. Ovarian tumors can originate from epithelial cells, stromal cells, and germ cells. The most common form epithelial ovarian cancer (EOC) is composed of five diverse main histological subtypes on the basis of distinctive histological and genetic characteristics including: low-grade serous (LGSOC), endometrioid (ENOC), high-grade serous (HGSOC), clear cell (CCOC), and mucinous (MOC), and [1, 5, 6, 9–11].

The outcomes of ovarian cancer treatment are dependent on early diagnosis, appropriate surgery, and effective systemic therapy [1, 12]. The clinical management of ovarian cancer includes debulking surgery, combination chemotherapy, radiation therapy, and other adjuvant therapies such as angiogenesis inhibitors in patients with suboptimally debulked and stage IV cancer, folate receptor targeting, and immunotherapy [1, 5, 6, 9–11]. A recent major progress has been made in maintenance therapy by including poly (ADP-ribose) polymerase (PARP) inhibitors in recurrent diseases and in a frontline regime among patients having *BRCA1*/*BRCA2* mutations [13, 14]. Nonetheless, combination therapy with platinum-based drugs (e.g., cisplatin, carboplatin, or oxaliplatin) and paclitaxel is the first-line systemic therapy [1]. Even though the five-year survival rate has improved steadily for the past two decades, the OC overall cure rate hovers around ~30% [5, 15]. Many patients have recurrence within 12–24 months and eventually succumb to chemotherapy-resistant cancer [5, 15]. Thus, there is an urgent unmet clinical need to identify new and effective anticancer agents to treat chemoresistant ovarian cancer.

Repurposing previously-approved drugs for cancer therapy is an appealing, safe and cost-effective approach to cancer drug discovery [16, 17]. Mebendazole (MBZ) is among the drugs with promising repurposing potential [17]. Approved by the US FDA to treat parasitic infections, MBZ has a long and favorable track-record of biosafety profiles in humans and in animal models [17]. We have recently demonstrated that MBZ can enhance cisplatin’s anticancer activities in head and neck squamous cell carcinoma (HNSCC) cells [18]. Other studies also indicate that MBZ and/or its derivatives exhibited anticancer activities in several types of human cancers [19–35]. Nonetheless, few studies have been carried out thus far to elucidate whether MBZ can effectively overcome chemoresistance and/or sensitize chemoresistant cancer cells to chemotherapeutics such as cisplatin.

In this study, we investigate whether MBZ overcomes cisplatin resistance and sensitizes chemoresistant cells to cisplatin in human ovarian cancer cells. We first established and characterized two stable and robust cisplatin-resistant (CR) human ovarian cancer lines and demonstrated that MBZ markedly inhibited cell proliferation, suppressed cell wounding healing/migration, and induced apoptosis in both parental and CR cells at very low micromole range. Mechanistically, MBZ was shown to inhibit multiple cancer-associated signaling pathways including ELK/SRF, NFKB, MYC/MAX, and E2F/DP1 in cisplatin-resistant ovarian cancer cells. We further demonstrated that MBZ synergized with cisplatin to suppress cell proliferation, induce cell apoptosis, and blunt tumor growth in the xenograft tumor model of human cisplatin-resistant ovarian cancer cells. Collectively, our results suggest that MBZ may be repurposed as a synergistic sensitizer of cisplatin in treating chemoresistant human ovarian cancer.

## RESULTS

### OVCAR8CR and SKOV3CR are stable cisplatin-resistant (CR) ovarian cancer cell lines with characteristics of chemoresistance

Since there has been a limited availability of human ovarian cancer lines that are stably resistant to cisplatin in the cancer research community [36–38], we sought to establish stable cisplatin-resistant human ovarian cancer lines from two commonly-used OVCAR8 and SKOV3 cell lines through a cisplatin dose-escalating selection process. When exponentially growing OVCAR8 and SKOV3 cells were initially treated with 0.5 μM cisplatin, vast majority of the cells were killed by cisplatin while a small fraction of the cells survived the selection. The viable cells were subsequently grown up and subjected to another round of selection with 0.5 μM cisplatin. Such selection scheme was carried out by escalating cisplatin concentrations gradually to 1.0 μM, 1.5 μM, 2.0 μM, 3.0 μM and 5.0 μM, yielding the stable cisplatin-resistant cell lines, namely OVCAR8CR and SKOV3CR.

**Figure 1:**
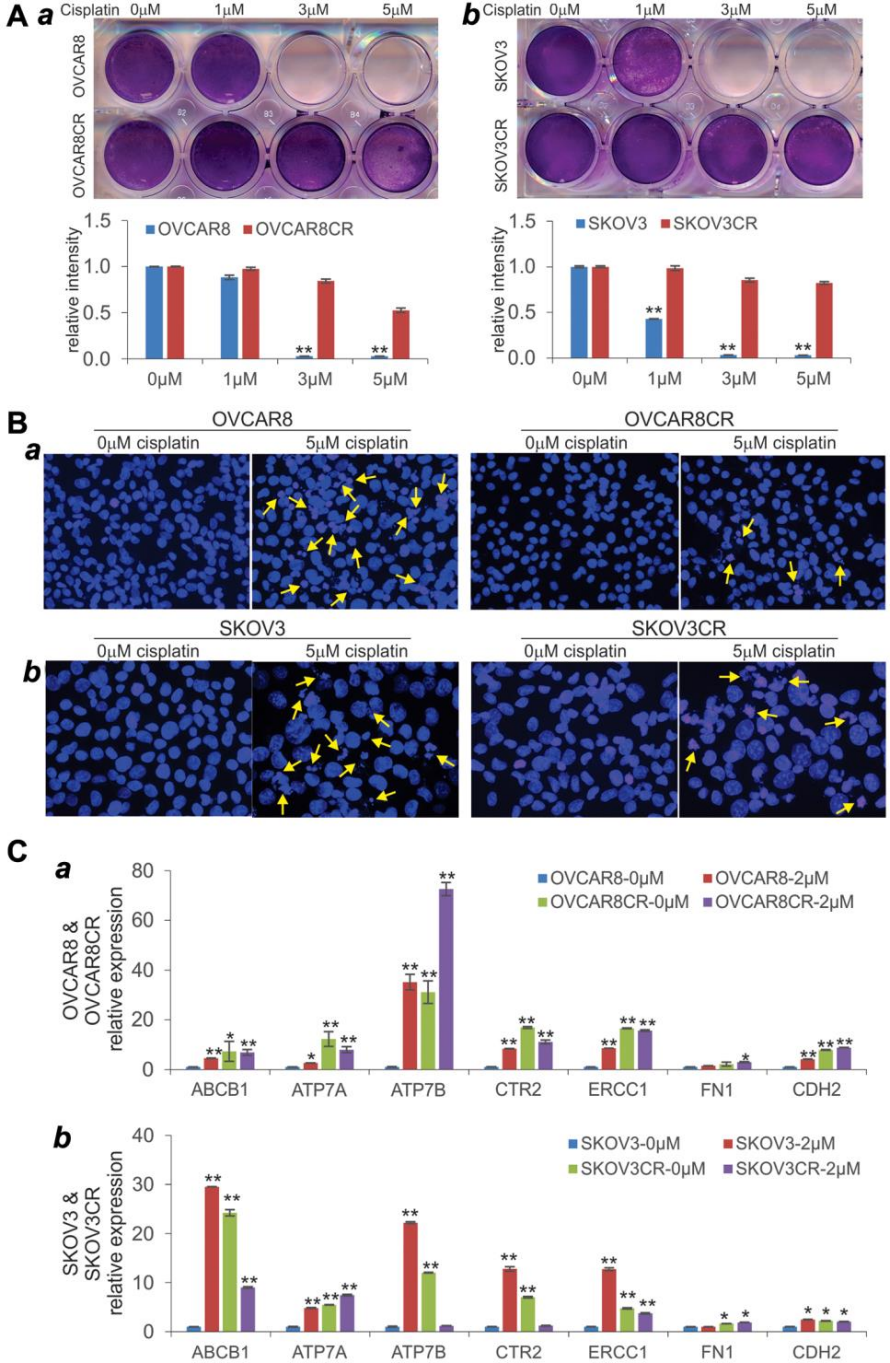
**Characterization of human cisplatin-resistant (CR) ovarian cancer cell lines.** (**A**) Crystal violet cell viability assay. Subconfluent two CR lines OVCAR8CR (***a***) and SKOV3CR (***b***), and the respective parental counterparts OVCAR8 (***a***) and SKOV3 (***b***) were treated with the indicated concentrations of cisplatin. At 72 h post treatment, cells were fixed and subjected to crystal violet staining. Representative results are shown. The stained cells were dissolved and measured quantitatively for optical absorbance. ^**^p < 0.01, compared with that of the parental cells treated with 0 μM cisplatin (or DFM solvent control) group. (**B**) Cell apoptosis assay. Subconfluent OVCAR8 (***a***), OVCAR8CR (***b***), SKOV3CR (***c***) and SKOV3CR (***d***) were treated with 0 or 5 μM cisplatin. At 72 h post treatment, cells were collected, fixed and stained with Hoechst33258 and examined under a fluorescence microscope. Representative images are shown. Representative apoptotic cells are indicated by arrows. (**C**) Expression of the chemoresistance-associated genes in the two cisplatin-resistant human ovarian cancer lines. Subconfluent two CR lines OVCAR8CR (***a***) and SKOV3CR (***b***), and the respective parental counterparts OVCAR8 (***a***) and SKOV3 (***b***) were treated with 0 or 2 μM cisplatin. At 48 h after treatment, total RNA was isolated and subjected to qPCR analysis of the indicated genes. *GAPDH* was used as a reference gene. All assays were done in triplicate. ^*^p < 0.05, ^**^p < 0.01, compared with that of the parental cells treated with 0 μM cisplatin (i.e., DFM solvent control) group.

As shown in Figure 1A, both OVCAR8CR and SKOV3CR lines effectively survived cisplatin treatment at as high as 5.0 μM, compared with their parental lines OVCAR8 (Figure 1A-*a*) and SKOV3 (Figure 1A-*b*), respectively. We also demonstrated that both OVCAR8CR and SKOV3CR cells were resistant to cisplatin-induced apoptosis, compared with their parental lines OVCAR8 (Figure 1B-*a*) and SKOV3 (Figure 1B-*b*), respectively. Furthermore, we analyzed the expression of a panel of chemoresistance-associated genes in the two cisplatin-resistant human ovarian cancer lines and found that most of them were up-regulated by cisplatin, and almost all of them were elevated in OVCAR8CR (Figure 1C-*a*) and SKOV3CR (Figure 1C-*b*), respectively. Based on the cell viability analysis, the calculated IC_50_ values for parental and resistant lines are as follows: OVCAR8, 1.50 μM; OVCAR8CR, 5.17 μM; SKOV3, 2.84 μM; and SKOV3CR, 122.26 μM. Collectively, these results demonstrate that both OVCAR8CR and SKOV3CR confer robust cisplatin resistance and exhibit the molecular and cellular characteristics of chemoresistant cancer cells.

### Mebendazole (MBZ) inhibits the cell viability and proliferation of human cisplatin-resistant ovarian cancer cells

**Figure 2:**
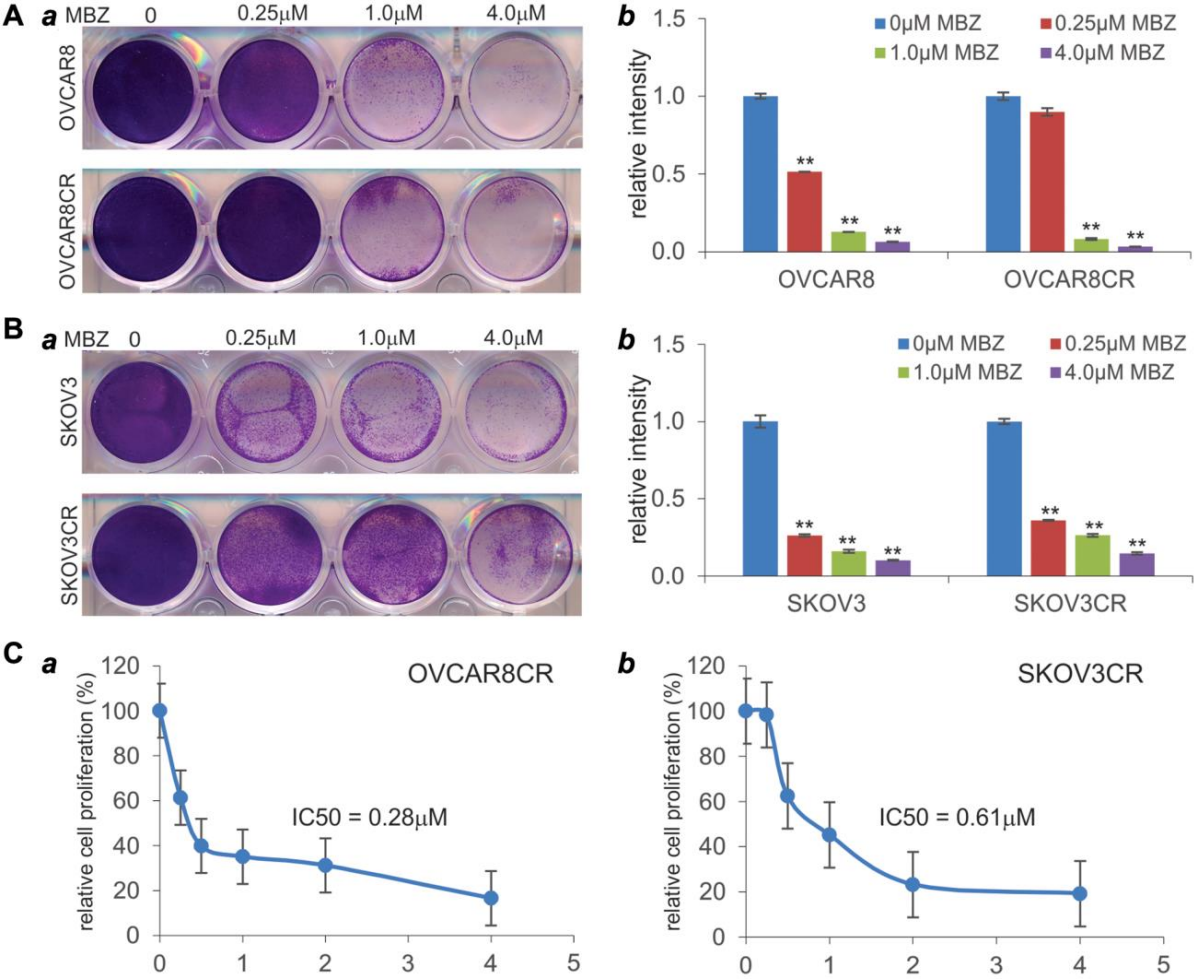
**Mebendazole (MBZ) effectively inhibits the cell viability and proliferation of human CR ovarian cancer lines.** (**A** and **B**) Crystal violet cell viability assay. Subconfluent OVCAR8 and OVCAR8CR (**A**) and SKOV3 and SKOV3CR (**B**) were treated with the indicated concentrations of MBZ. At 72 h post treatment, cells were fixed and subjected to crystal violet staining (***a***). Representative results are shown. The stained cells were dissolved and measured quantitatively for optical absorbance (***b***). ^**^p < 0.01, compared with that of the respective cells treated with 0 μM MBZ (or DMSO solvent control) group. (**C**) WST-1 cell proliferation assay. Subconfluent OVCAR8CR (***a***) and SKOV3CR (***b***) cells were seeded in 96-well plates and treated with MBZ at the indicated concentrations. At 72 h after treatment, the WST-1 regent (Takara BIO USA, Inc.) was added to each well, and incubated for 2 h prior to the absorbance reading of each well. The IC_50_ values were calculated by using the AAT Bioquest online tools. All assay conditions were done in triplicate.

We next tested whether MBZ was capable of inhibiting the cell viability of the CR ovarian cancer cells. When both OVCAR8CR and its parental OVCAR8 cells were treated with MBZ, we found that the numbers of viable cells drastically decreased at as low as 1.0 μM of MBZ, and completely eliminated at 4.0 μM of MBZ (Figure 2A-*a*). In fact, a quantitative analysis indicates that MBZ significantly decreased the cell viability of the parental OVCAR8 cells at as low as 0.25 μM (Figure 2A-*b*). Similarly, MBZ was shown to effectively decrease the cell viability of both SKOV3 and SKOV3CR cells at as low as 0.25 μM (Figure 2B-*ab*), although more surviving cells were found in the SKOV3 and SKOV3CR treated with 4 μM MBZ, than that found in the OVCAR8CR and OVCAR8 cells under the same treatment conditions (Figures 2A vs. 2B).

We further examined the effect of MBZ on the cell proliferative activity of the CR ovarian cancer cells. MBZ was shown to inhibit cell proliferation of OVCAR8CR cells in a dose-dependent fashion with an IC_50_ at 0.28 μM (Figure 2C-*a*). Similarly, MBZ also effectively inhibited cell proliferation of SKOV3CR cells in a dose-dependent fashion with an IC_50_ at 0.61 μM (Figure 2C-*b*). Collectively, these results demonstrate that MBZ may be able to overcome cisplatin resistance in the CR ovarian cancer cells by inhibiting cell viability and proliferative activity of ovarian cancer cells.

### MBZ inhibits cell wound healing/migration and induces apoptosis in cisplatin-resistant ovarian cancer cells

**Figure 3:**
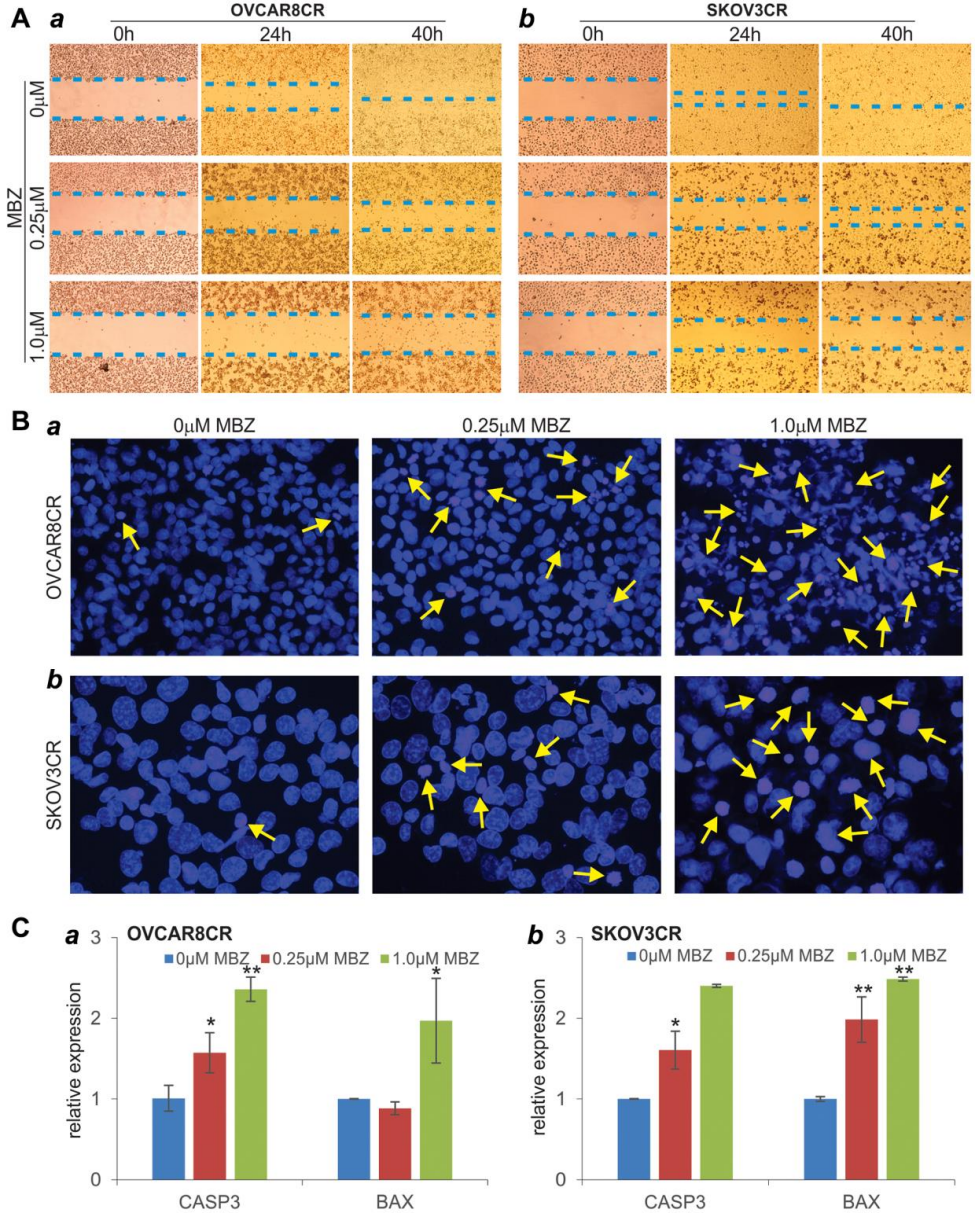
**MBZ effectively inhibits cell wound healing/migration and induces apoptosis in the human CR ovarian cancer cells.** (**A**) Cell wounding/migration assay. Freshly subconfluent OVCAR8CR (***a***) and SKOV3CR (***b***) cells were wounded with micro-pipette tips and treated with the indicated concentrations of MBZ. The wounding gaps were recorded at 0 h, 24 h and 40 h after MBZ treatment. Each assay condition was done in triplicate. Representative results are shown (**B**) Cell apoptosis assay. Subconfluent OVCAR8CR (***a***) and SKOV3CR (***b***) cells were treated with the indicated concentrations of MBZ. At 72 h after treatment, cells were collected, fixed and stained with Hoechst 33258 and examined under a fluorescence microscope. Representative images are shown. Representative apoptotic cells are indicated by arrows. (**C**) The expression of apoptosis-inducing genes. Subconfluent OVCAR8CR (***a***) and SKOV3CR (***b***) cells were treated with the indicated concentrations of MBZ for 48 h. Total RNA was isolated and subjected to qPCR analysis of the expression of CASP3 and BAX. GAPDH was used as the reference gene. All assays were done in triplicate. ^*^p < 0.05, ^**^p < 0.01, compared with that of the cells treated with 0 μM MBZ (i.e., DMSO solvent control) group.

We also tested the effect of MBZ on cell wound healing/migration of cisplatin-resistant ovarian cancer cells. MBZ was shown to effectively inhibit the wound closure of the injured OVCAR8CR cells in a dose-dependent manner (Figure 3A-*a*). In fact, nearly 80% of the wound gap remained open at 40h after treated with 1 μM MBZ, while the control group was completely healed (Figure 3A-*a*). Similar dose-dependent inhibitory effect of MBZ was observed on the wound closure of the injured SKOV3CR cells (Figure 3A-*b*). These results indicate that MBZ can inhibit cell migration and proliferation of the CR ovarian cancer cells at low micromole concentrations.

We next analyzed whether MBZ induced cell apoptosis in cisplatin-resistant ovarian cancer cells. When the OVCAR8CR and SKOV3CR cells were treated with 0.25 μM and 1 μM MBZ, Hoechst 33258 staining indicated that the numbers of apoptotic cells significantly increased in MBZ-treated cells, compared with that of the control group (Figure 3B-*ab*). We further analyzed the effect of the expression of pro-apoptotic genes BAX and CASP3, and found that upon MBZ treatment (especially at 1 μM MBZ) both BAX and CASP3 expression levels were significantly elevated in the OVCAR8CR and SKOV3CR cells (Figure 3C-*ab*). Collectively, these results suggest that MBZ may overcome cisplatin resistance, at least in part, by inhibiting cell migration/proliferation and inducing apoptosis in chemoresistant ovarian cancer cells.

### MBZ inhibits multiple cancer-associated signaling pathways in human cisplatin-resistant ovarian cancer cells

**Figure 4:**
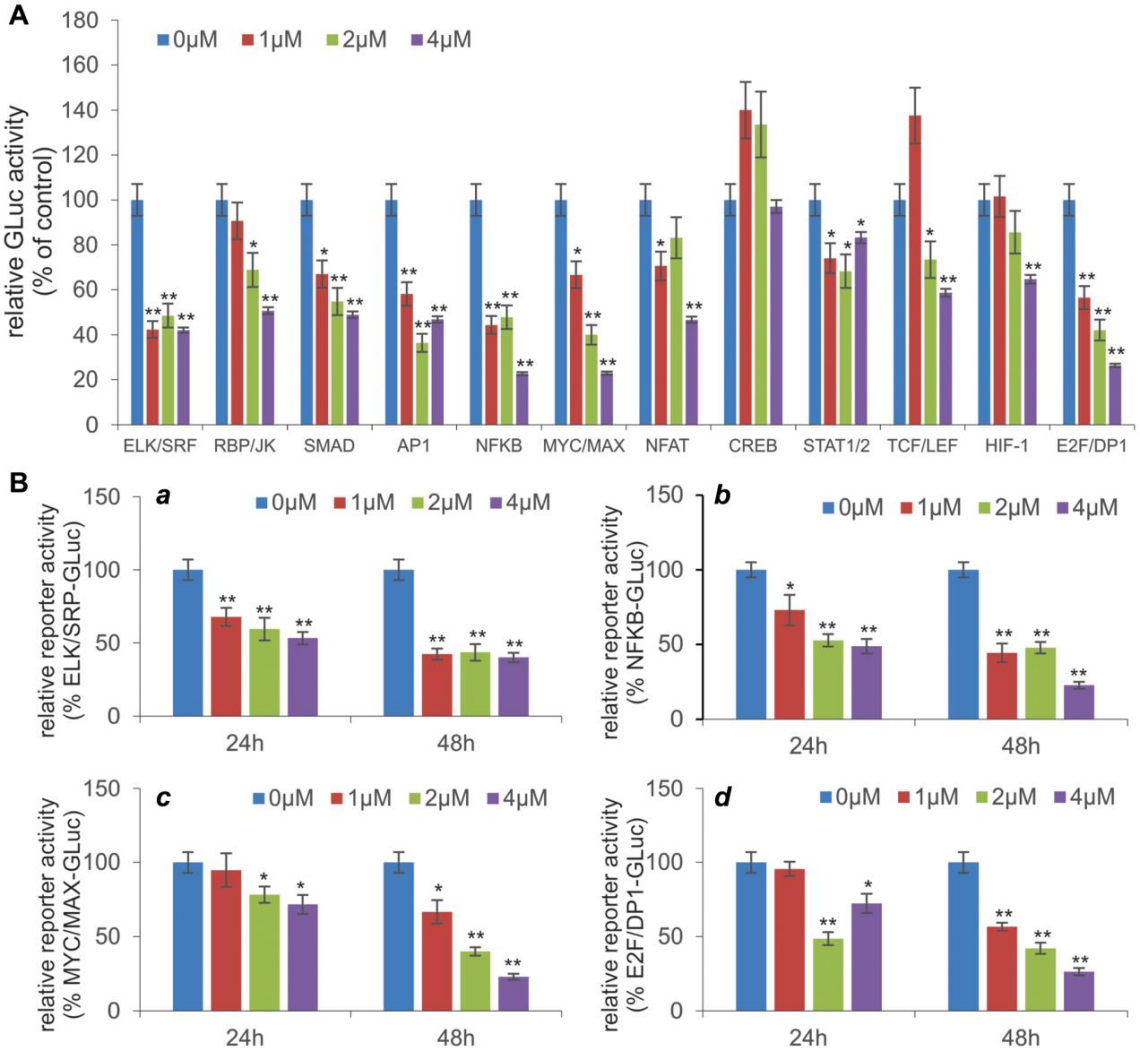
**MBZ inhibits multiple cancer-associated signaling pathways in human CR ovarian cancer cells.** (**A**) Effect of MBZ on the 12 cancer-associated pathways in CR human ovarian cancer cells. Subconfluent SKOV3CR cells were transfected with the Gaussia luciferase reporter plasmids for the 12 cancer-associated pathways. At 24 h post transfection, the cells were treated with the indicated concentrations of MBZ for additional 48 h. The culture medium was collected for Gaussia luciferase assay using the Gaussia Luciferase Assay Kit (GeneCopoeia, Rockville, MD). Each assay condition was done in triplicate. ^*^p < 0.05, ^**^p < 0.01, compared with that of the cells treated with 0 μM MBZ (i.e., DMSO solvent control) group. (**B**) MBZ inhibits five cancer-related pathways in dose- and time-dependent manners. The selected five pathway reporter plasmids ELK/SRP (***a***), NFKB (***b***), MYC/MAX (***c***), and E2F/DP1 (***d***) were transfected into SKOV3CR cells as described in (**A**). The transfected cells were treated with the indicated concentrations of MBZ for 24 h or 48 h, followed by Gaussia Luciferase activity assays. Each assay condition was done in triplicate. ^*^p < 0.05, ^**^p < 0.01, compared with that of the cells treated with 0 μM MBZ (i.e., DMSO solvent control) group.

Mechanistically, several studies have reported that MBZ exerts its anticancer activity by regulating numerous cellular pathways. However, it is not clear whether MBZ can overcome cisplatin-based chemoresistance in ovarian cancer cells through similar mechanisms. Thus, we examined the effect of MBZ on the 12 cancer-related signaling pathways in the cisplatin-resistant ovarian cancer cells. When the pathway reporters were transfected into those cells and treated with various concentrations of MBZ, we found that 11 of the 12 pathways, especially ELK/SRF, NFKB, MYC/MAX, E2F/DP1, TGF/SMAD and AP1 pathway reporters, were effectively inhibited by MBZ in a dose-dependent fashion although the CREB pathway reporter was seemingly activated at 1 μM and 2 μM MBZ (Figure 4A), suggesting that MBZ may exert its anticancer and anti-chemoresistance activities in ovarian cancer cells by inhibiting multiple cancer-related signaling pathways.

We further analyzed the dose-dependent and time-dependent inhibitory effect of MBZ on the four most impacted pathways, ELK/SRF, NFKB, MYC/MAX, and E2F/DP1. We found that the ELK/SRF reporter activities were significantly inhibited by MBZ in dose-dependent and time-dependent fashion (Figure 4B-*a*). Similar inhibitory effects by MBZ were observed for the NFKB (Figure 4B-*b*), MYC/MAX (Figure 4B-*c*), and E2F/DP1 (Figure 4B-*d*) reporter activities, suggesting that MBZ may exert profound inhibitory effects on cell proliferation pathways in cisplatin-resistant ovarian cancer cells.

### MBZ synergizes with cisplatin to inhibit cell proliferation, induce cell apoptosis, and suppress tumor growth in the xenograft model of human cisplatin-resistant ovarian cancer cells

**Figure 5:**
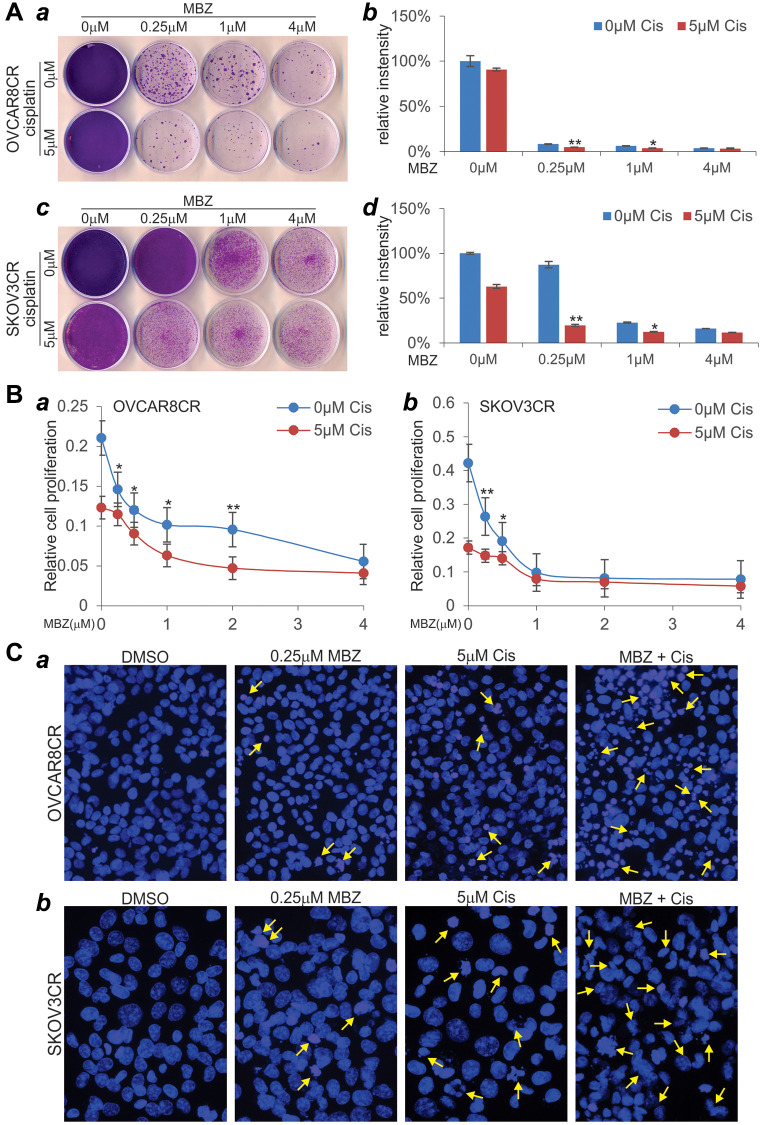
**MBZ synergizes with cisplatin to inhibit cell proliferation and induce apoptosis in the human CR ovarian cancer cells.** (**A**) Colony formation and crystal violet cell viability assay. Subconfluent OVCAR8CR (***a***, ***b***) and SKOV3CR (***c***, ***d***) cells were treated with MBZ and cisplatin at the indicated concentrations. At 72 h post treatment, the cells were replated for colony formation for 10 days, followed by crystal violet staining (***a***, ***c***). Each assay condition was done in triplicate. Representative results are shown (***a***, ***c***). The stained cells were dissolved in acetic acid and quantitatively measured for optical absorbance (***b***, ***d***). ^*^p < 0.05 ^**^p < 0.01, when compared with that of the respective 0 μM cisplatin groups. (**B**) WST-1 cell proliferation assay. Subconfluent OVCAR8CR (***a***) and SKOV3CR (***b***) cells were seeded into 96-well cell culture plates, and treated with DMSO, cisplatin and/or MBZ at the indicated concentrations. At 72 h post treatment, WST-1 working mix was added to each well and incubated for 2h prior to absorbance reading at 450nm. Each assay condition was done in triplicate. ^*^p < 0.05 ^**^p < 0.01, when compared with that of the respective 0 μM cisplatin groups. (**C**) Cell apoptosis assay. Subconfluent OVCAR8CR (***a***) and SKOV3CR (***b***) cells were seeded into 6-well cell culture plates, and treated with DMSO, 5 μM cisplatin (Cis) and/or 0.25 μM MBZ. At 72 h, the cells were collected, fixed, stained with Hoechst33258, and examined under a fluorescence microscope. Representative images are shown. Representative apoptotic cells are indicated by arrows.

We next tested whether MBZ could sensitize the cisplatin-resistant ovarian cancer cells to cisplatin. As demonstrated earlier, although 5 μM cisplatin alone in OVCAR8CR cells did not significantly impact cell viability, the presence of MBZ at a concentration as low as 0.25 μM drastically diminished cell viability and reduced cell colonies (Figure 5A-*ab*). In SKOV3CR cells, even though 5 μM cisplatin alone did affect cell viability, the presence of MBZ at a concentration as low as 0.25 μM markedly reduced cell viability and formed significantly fewer colonies (Figure 5A-*cd*). Interestingly, MBZ concentrations increases (from 0.25 μM to 4 μM) did not significantly enhance cisplatin-mediated cytotoxicity (Figure 5A-*cd*).

We also conducted WST-1 assay to investigate the effect of MBZ on cell proliferation. Even though 5 μM cisplatin alone inhibited cell proliferation, the presence of MBZ, at a concentration as low as 0.25 μM, significantly inhibited the cell proliferation of OVCAR8CR cells (Figure 5B-*a*). Similarly, while 5 μM cisplatin alone could inhibit cell proliferation, the presence of MBZ, at a concentration as low as 0.25 μM, significantly inhibited the cell proliferation of SKOV3CR cells, although higher concentrations of MBZ did not exhibit more inhibitory effect on cell proliferation (Figure 5B-*b*).

We further analyzed the effect of MBZ on cisplatin-induced apoptosis in cisplatin-resistant ovarian cancer cells. Even though 0.25 μM MBZ or 5 μM cisplatin alone caused detectable cell apoptosis in OVCAR8CR cells, the combination of both led a significant increase in apoptotic cells (Figure 5C-*a*). Similarly, the combination of 0.25 μM MBZ and 5 μM cisplatin caused a marked increase of cell apoptosis in SKOV3CR cells, compared that treated with either drug alone (Figure 5C-*b*). Taken together, these *in vitro* results strongly suggest that MBZ may sensitize the chemoresistant ovarian cancer cells to cisplatin.

**Figure 6:**
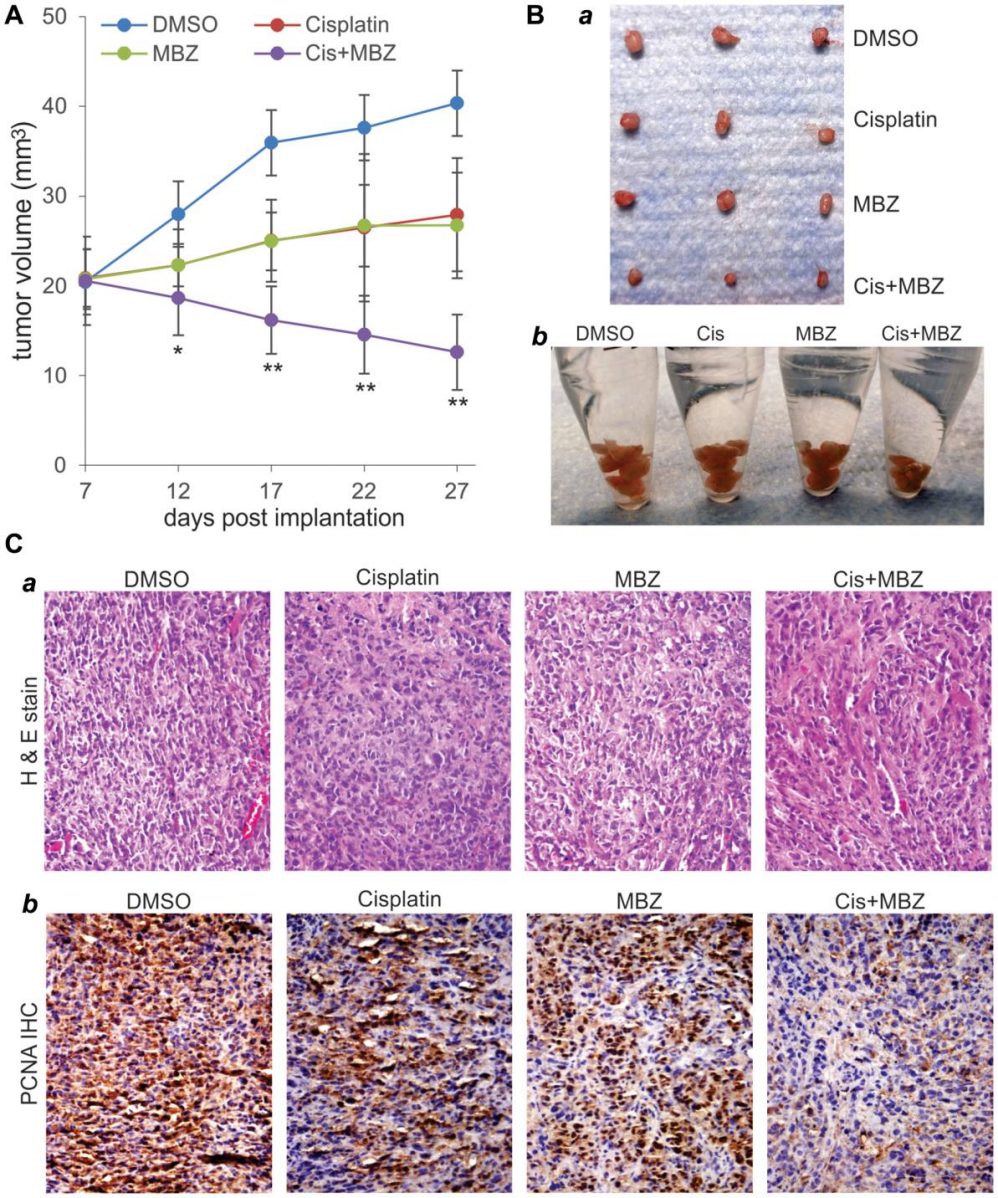
**MBZ and cisplatin synergistically inhibit tumor growth in the xenograft tumor model of human CR ovarian cancer cells.** Exponentially growing SKOV3CR cells were collected and subcutaneously injected into the flanks of athymic nude mice (*n* = 6 per group). At 7 days post injection, the mice were randomly divided into four groups, and treated with DMSO, MBZ, and/or Cisplatin (Cis) for three weeks. Tumor growth was monitored and average tumor growth was calculated. (**A**) Representative retrieved tumor masses were photographed individually (**B**-***a***) or pooled in Eppendorf tubes. (**B**-***b***) The retrieved tumor masses were subjected to H & E staining (**C**-***a***), and anti-PCNA immunochemical staining (**C**-***b***). Minus primary antibody and control IgG were used as negative controls. Representative results are shown.

Lastly, we analyzed the potential synergistic effect between MBZ and cisplatin on *in vivo* tumor growth. Using a xenograft tumor model of SKOV3CR cells, we found that, even though MBZ or cisplatin alone slowed down tumor growth, the combination of MBZ and cisplatin drastically suppressed the xenograft tumor growth, compared with that treated with either drug alone (Figure 6A), which was confirmed by gross examining of the retrieved tumor masses (Figure 6B-*ab*). Histologic evaluation indicates that xenograft tumors treated with MBZ and cisplatin alone or in combination exhibited significant necrosis with reduced cell numbers, compared with that of the DMSO control group (Figure 6C-*a*). Immunohistochemical staining with a PCNA antibody demonstrate that cell proliferation was drastically inhibited in the xenograft tumors treated with MBZ or cisplatin, while the combination of MBZ and cisplatin led to the greatest decrease in cell proliferation in the retrieved tumor masses (Figure 6C-*b*). Thus, these *in vivo* finding further demonstrate that MBZ and cisplatin could synergistically inhibit tumor growth in the xenograft tumor model of human CR ovarian cancer cells.

## DISCUSSION

Any significant improvement in long-term survival of ovarian cancer patients hinges on translating our understanding of molecular pathogenesis of ovarian cancer into precision treatment strategies, devising novel methods for screening or early detection, and developing new therapeutics [5, 6, 9–11, 39]. The use of stable platinum or paclitaxel-resistant ovarian cancer lines is critical to the understanding and overcoming of ovarian cancer chemoresistance in order to improve the long-term survival of ovarian cancer patients. Histologically, there have been a rather limited availability of human ovarian cancer lines that are stably resistant to cisplatin in the cancer research community [37, 38]. A commonly-used cisplatin resistant ovarian cancer line A2780^CP^ or A2780/CP70 was first reported in 1988 [36–38], although other cisplatin resistant lines were later established from lesser known cell lines or treated patients [40–42]. Here, we sought to establish stable cisplatin resistant human ovarian cancer lines from two commonly-used OVCAR8 and SKOV3 cell lines through a cisplatin dose-escalating selection process. We demonstrated that both OVCAR8CR and SKOV3CR lines can confer robust and stable resistance to at least 5 μM cisplatin in culture. Furthermore, we demonstrated that the OVCAR8CR and SKOV3CR lines share similar biological characteristics to other chemoresistance, especially platinum-resistance, cancer lines as the expression of representative chemoresistance-related genes were effectively up-regulated in both cell lines. Thus, OVCAR8CR and SKOV3CR lines should be a valuable research resource for studying cisplatin resistance in ovarian cancer.

Repurposing of drugs approved for other indications is an attractive strategy to develop new anti-cancer agents in a cost-effective and less time-consuming fashion [16, 43]. In recent years, several members of the synthetic anthelminthic benzimidazole family, such as mebendazole (MBZ), fenbendazole (FBZ), flubendazole, and albendazole (ABZ), have shown great promise to be repurposed as anticancer agents [16, 17, 28]. This family of anti-parasitic drugs has been used to treat pinworm and other helminthic infections in humans and animals with excellent safety records over decades [28]. Among them, MBZ is particularly appealing as it meets the desirable characteristics for anticancer agent repurposing: excellent and proven biosafety profile, favorable pharmacokinetics for reaching therapeutic concentrations at disease site, ease of delivery, and low cost [17, 28]. We demonstrated that MBZ exerted more potent anti-proliferation activity than cisplatin in human HNSCC cells, and effectively inhibited cell proliferation, cell cycle progression and cell migration, and induced apoptosis in HNSCC cells [18]. Other studies indicate that MBZ and its derivatives exerted potent anticancer effect in non-small cell lung cancer [19, 20], adrenocortical carcinoma [22], medulloblastoma [28], melanoma [21], leukemia and myeloma [24], glioblastoma multiform [23, 34], colon cancer [26], cholangiocarcinoma [27], breast cancer [25, 29], gastric cancer [30], mouse hepatoma [31], and thyroid cancer [32]. In this study, our results are the first to demonstrate that MBZ can overcome cisplatin resistance and synergize with cisplatin in inhibiting cell proliferation and inducing apoptosis in cisplatin-resistant ovarian cancer cells.

Mechanistically, we examined the effect of MBZ on the 12 cancer-related signaling pathways in the cisplatin-resistant ovarian cancer cells. When the pathway reporters were transfected into those cells and treated with various concentrations of MBZ, we found that 11 of the 12 pathways, especially ELK/SRF, NFKB, MYC/MAX, E2F/DP1, TGF/SMAD and AP1 pathway reporters, were effectively inhibited by MBZ in a dose-dependent fashion although the CREB pathway reporter was seemingly activated by MBZ, suggesting that MBZ may exert its anticancer and anti-chemoresistance activities in ovarian cancer cells by inhibiting multiple cancer-related cell proliferation pathways in cisplatin-resistant ovarian cancer cells. These results are consistent with our recent studies, in which we demonstrated that MBZ modulated the cancer-associated pathways including ELK1/SRF, AP1, STAT1/2, MYC/MAX, and synergized with cisplatin in suppressing cell proliferation and inducing apoptosis of human HNSCC cells [18].

Several studies also indicate that MBZ may exert its anticancer activity by regulating multiple cellular pathways [17]. It was reported that MBZ in medulloblastoma inhibited tumor angiogenesis [28]. We and others found that MBZ effectively induced mitotic arrest and apoptosis by depolymerizing tubulin in cancer cells [18, 20, 22]. A recent study showed that MBZ’s anticancer activity was associated with p53-independent induction of p21 and tubule depolymerization in ovarian cancer cells [35]. Interestingly, an *in silico* molecular target screening predicted MBZ as a potent MAPK14 inhibitor [44], while MBZ was shown to activate MEK-ERK pathway in monocytes and macrophages [45]. MBZ was shown to promote the terminal differentiation of the HNSCC CAL27 cells and keratinization of CAL27-derived xenograft tumors [18], and MBZ was also shown to function as differentiation therapy for human acute myeloid leukemia (AML) cells [46]. MBZ was shown to suppress cell proliferation and/or induce apoptosis through inactivating C-MYC pathway in malignant ascites cells and gastric cancer cells [47], inhibiting USP5/c-Maf axis in multiple myeloma [48] or TRAF2- and NCK-interacting kinase (TNIK) in colon cancer cells [49], and downregulating XIAP expression in melanoma cells [50]. MBZ was also identified as a hedgehog signaling inhibitor [51]. Interestingly, MBZ was shown to sensitize cancer cells to ionizing radiation [52] and potentiate radiation therapy for triple-negative breast cancer cells [53]. Nonetheless, the exact mechanism through which MBZ exerts anticancer activities and overcomes cancer chemoresistance remains to be thoroughly investigated.

## CONCLUSIONS

We investigated whether MBZ could overcome cisplatin resistance and sensitize chemoresistant ovarian cancer cells to cisplatin. Using our established and characterized two CR human ovarian cancer lines, we demonstrated that MBZ markedly inhibited cell proliferation, suppressed cell wounding healing/migration, and induced apoptosis in both parental and CR cells. Mechanistically, MBZ was shown to inhibit multiple cancer-related signal pathways including ELK/SRF, NFKB, MYC/MAX, and E2F/DP1 in cisplatin-resistant ovarian cancer cells. We further demonstrated that MBZ synergized with cisplatin to inhibit cell proliferation, induce cell apoptosis, and suppress tumor growth in the xenograft model of human CR ovarian cancer cells. Taken together, our findings strongly suggest that MBZ may be repurposed as a synergistic sensitizer of cisplatin in treating chemoresistant human ovarian cancer, which should be thoroughly investigated in clinical trials.

## MATERIALS AND METHODS

### Cell culture and chemicals

Human ovarian cancer cell lines OVCAR8 and SKOV3 were kindly provided by Dr. Ernest Lengyel of The University of Chicago. All cells were cultured in DMEM with 10% fetal bovine serum (FBS, Gemini Bio-Products, West Sacramento, CA), 100 U/mL penicillin, and 100 μg/mL streptomycin at 37°C in 5% CO_2_ as previously reported [54–57]. Research grades of cisplatin (Cis) and mebendazole (MBZ) were purchased from Selleckchem (Houston, TX). Other chemicals were purchased either from Sigma-Aldrich (St. Louis, MO) or from Thermo Fisher Scientific (Waltham, MA).

### Establishment of the cisplatin-resistant (CR) human ovarian cancer lines OVCAR8CR and SKOV3CR

Exponentially growing human ovarian cancer cell lines OVCAR8 and SKOV3 were treated with 0.5 μM cisplatin (dissolved in dimethylformamide or DMF; the cisplatin stock solution was aliquoted and kept at –80°C) for 72 h. Viable cells were replated and grown up, followed by one more round treatment of 0.5 μM cisplatin. Similar selection scheme was carried out by escalating cisplatin concentrations gradually to 1.0 μM, 1.5 μM, 2.0 μM, 3.0 μM and 5.0 μM, yielding the stable and robust cisplatin-resistant cell lines that are designated as OVCAR8CR and SKOV3CR. These lines were further characterized in the reported work.

### Crystal violet staining

Cell viability was assessed by using the crystal violet staining as described [58–62]. Experimentally, subconfluent ovarian cancer cells were seeded in 6-well or 12-well cell culture plates, and treated with various concentrations of cisplatin and/or MBZ. At 72 h post treatment, the cells were washed with PBS and then stained with 0.5% crystal violet/formalin solution. The stained cells were washed with tape water, air dried and scanned for image documentation. For quantitative analysis, the stained cells were dissolved in 10% acetic acid, followed by measuring absorbance at 570–590nm. Each assay condition was carried out in triplicate.

### RNA isolation and touchdown-qPCR (TqPCR)

Subconfluent cells were treated with various conditions for 48h. Total RNA was isolated by using the NucleoZOL reagent (Takara Bio USA Inc.), and subjected to reverse transcription (RT) reactions as previously described [63–66]. The RT cDNA products were used as PCR templates. The primers for the genes of interest were designed by using Primer3 Plus program (Supplementary Table 1). TqPCR reactions were carried out by using SYBR Green-based Forget-Me-Not™ qPCR Master Mix (Biotium Inc., Hayward, CA) on a CFX-Connect unit (Bio-Rad Laboratories, Hercules, CA) as described [67–70]. Relative gene expression was normalized to *GAPDH* by using the 2^−∆∆Ct^ method. All qPCR reactions were done in triplicate.

### Cell colony formation assay

Subconfluent ovarian cancer cells were plated in 12-well cell culture plates and treated with various concentrations of cisplatin and/or MBZ. At 72 h after treatment, cells were replated into 30mm cell culture dishes, and cultured in compete DMEM medium for additional 10 days. The colonies were then stained with 0.5% crystal violet/formalin solution and scanned for image documentation. For quantitative analysis, the stained cells were dissolved in 10% acetic acid and measured for absorbance at 570–590nm. Each assay condition was carried out in triplicate.

### WST-1 cell proliferation assay

Cell proliferation was quantitatively determined with Premixed WST-1 Reagent (Takara Bio USA Inc., Mountain View, CA) as described [54, 71–75]. Experimentally, OVCAR8CR and SKOV3CR cells were seeded into 96-well culture plates, and treated with varied concentrations of cisplatin and/or MBZ. At 72h post treatment, the freshly prepared WST-1 Working Mix were added into each well, and incubating at 37°C for 2 h before subjecting the plates to absorbance reading at 450nm using a microplate reader (BioTek EL800, Winooski, VT). The IC50 values were calculated by using the AAT Bioquest online tools. Each assay condition was done in triplicate.

### Cell wounding/migration assay

The cell wounding/migration experiments were performed as described [18, 76, 77]. Briefly, OVCAR8CR and SKOV3CR cells were seeded in 6-well plates and grown to 90% confluence. Monolayer cells were then scratched with sterile micro-pipette tips. At the indicated time points, the wound closure status at the same locations was recorded under a bright field microscope. Each assay condition was done in triplicate.

### Apoptosis analysis (Hoechst 33258 staining)

Hoechst 33258 staining assay was conducted as described [54, 78–80]. Specifically, exponentially growing human ovarian cancer cells were treated with varied concentrations of cisplatin and/or MBZ. At 72 h post treatment, the cells were collected and stained with the Magic Solution. Apoptotic cells were documented under a fluorescence microscope. Each assay condition was done in triplicate.

### Transfection and gaussia luciferase assay

The cancer-related pathway reporters and the Gaussia luciferase assay were previously described [81–83]. The tested cancer-related pathways included NFAT, HIF-1, TCF/LEF, E2F/DP1, ELK1/SRF, AP1, NFκB, SMAD, STAT1/2, RBP-JK, CREB, MYC/MAX pathway reporters. Briefly, exponentially growing SKOV3CR cells were seeded in 60mm cell culture dishes and transfected with 5.0 μg reporter plasmid DNA/dish of each reporter plasmid by using the PEI transfection reagent (Polysciences, Warrington, PA). At 24 h after transfection, the cells were replated into 24-well cell culture plates, and treated with various concentrations of MBZ. At 24 and/or 48 h after treatment, culture media were taken for Gaussia luciferase assays using the Gaussia Luciferase Assay Kit (GeneCopoeia, Rockville, MD). Each assay condition was done in triplicate.

### Xenograft tumor model of human ovarian cancer cells

The use and care of animals for the reported work were approved by the Institutional Animal Care and Use Committee. The xenograft tumor model of human ovarian cancer cells was established as previously described [71, 84–86]. Specifically, exponentially growing SKOV3CR cells were collected, resuspended in PBS at 1.5 × 10^8^ cells/ml, and injected subcutaneously into the flanks of athymic nude mice (Harlan Laboratories, 6–8 week old, female, 5 × 10^6^ cells per injection, and 4 injection sites per mouse). At 7 days post injection, the mice were divided into four groups (*n* = 6 per group): MBZ group (i.p. injection of 7.5 mg/kg body weight MBZ, once every two days); cisplatin group (i.p. 3mg/kg body weight cisplatin, once every two days); cisplatin/MBZ group (i.p. 7.5 mg/kg body weight MBZ, and 3mg/kg body weight cisplatin, once every two days); and DMSO control group. Tumor growth was monitored by caliper measurements at the indicated time points. Tumor volumes were calculated by using the formula (a × b^2^ × 0.52), whereas the “*a”* is the long dimension, and “*b*” being the short as previously described [87–89]. The nude mice were sacrificed at 27 days after cell injection. The tumor masses were retrieved for histologic evaluation and immunohistochemical analysis.

### Hematoxylin and eosin (H & E) staining & immunohistochemical (IHC) analysis

H & E histological analysis was carried out as described [90–94]. Briefly, the retrieved tumor masses were fixed with 10% PBS-buffered formalin and paraffin embedded. Serial sections were deparaffinized and subjected to H & E staining. Staining results were documented under a bright field microscope.

IHC analysis was carried out as described [57, 95–98]. Specifically, the tissue sections were deparafinized, rehydrated, and subjected to IHC staining with an anti-PCNA antibody (Santa Cruz Biotechnology, Dallas, TX). Minus primary antibody and control IgG were used as negative controls.

### Statistical analysis

All quantitative experiments were done in triplicate and/or in three independent batches. Data were expressed as mean ± standard deviation. Statistical significance was determined by one-way analysis of variance and the Student *t*-test. A *p*-value of <0.05 was defined as statistically significant.

## Supplementary Materials

Supplementary Table 1
