# Supplementary material for: Antiparasitic mebendazole (MBZ) effectively overcomes cisplatin resistance in human ovarian cancer cells by inhibiting multiple cancer-associated signaling pathways
Source: Aging (Albany NY). 2021 Jul 7;13(13):17407–27. doi: 10.18632/aging.203232 (PMC8312413; doi:10.18632/aging.203232)
Supplement: Supplementary Table 1 [file aging-13-203232-s001.pdf]

SUPPLEMENTARY TABLE

Supplementary Table 1. List of qPCR Primers.

GENE	Forward Primer	Reverse Primer
GAPDH	GTCAAGGCTGAGAACGGGAA	AAATGAGCCCCAGCCTTCTC
ABCB1	AGGGACTGAGCCTGGAGG	ACACGATGCCCAGGTGTG
ATP7A	GGGACTGGCCACTCCAAC	CCACTGGGGTTCCGTGAG
ATP7B	TTCGAGGCCAGCATTGCA	CGGCCTCTTGGTTGCTGA
CTR2	GAGGAACGTGCAGGCACT	TGCTGCAATGGCTTCCGA
ERCC1	GCAGAAACCAGCGGACCT	CTTTCTGAGGGCCCAGGC
FN1	CTGGGATGCTCCTGCTGT	GCCGCTGATGGTAGCTGT
CDH2	GTGCATGAAGGACAGCCTCT	GCCACTTGCCACTTTTCCTG
CASP3	TCCTAGCGGATGGGTGCT	GAAGAGGCAGGTGCAGGG
BAX	GCCCCACTAACTGTTGCATT	AGACTGCAGTGAGCCAAGGT
